# Challenges in the transition from resident to attending physician in general internal medicine: a multicenter qualitative study

**DOI:** 10.1186/s12909-022-03400-z

**Published:** 2022-05-02

**Authors:** Christine Roten, Christine Baumgartner, Stefanie Mosimann, Yonas Martin, Jacques Donzé, Felix Nohl, Simone Kraehenmann, Matteo Monti, Martin Perrig, Christoph Berendonk

**Affiliations:** 1 Department of General Internal Medicine, Inselspital, Bern University Hospital, University of Bern, Bern, Switzerland; 2 Faculty of Health, Medicine & Life Sciences, Maastricht University, 6229 ER Maastricht, The Netherlands; 3 Department of Infectious Diseases, Inselspital, Bern University Hospital, University of Bern, Bern, Switzerland; 4 Division of Medicine, Hôpital Neuchâtelois, Neuchâtel, Switzerland; 5 Department of General Internal Medicine, Regionalspital Emmental, Burgdorf, Switzerland; 6 Division of General Internal Medicine, Kantonsspital St. Gallen, St. Gallen, Switzerland; 7 Department of Internal Medicine and Medical Education Unit, Lausanne University Hospital and University of Lausanne, Lausanne, Switzerland; 8 Institute for Medical Education, University of Bern, Bern, Switzerland

**Keywords:** Postgraduate medical education, Hospital medicine, Transition, Residency, Attending physician, Professional competency, Qualitative research

## Abstract

**Background:**

The attending physician in general internal medicine (GIM) guarantees comprehensive care for persons with complex and/or multiple diseases. Attendings from other medical specialties often report that transitioning from resident to attending is burdensome and stressful. We set out to identify the specific challenges of newly appointed attendings in GIM and identify measures that help residents better prepare to meet these challenges.

**Methods:**

We explored the perceptions of 35 residents, attendings, and department heads in GIM through focus group discussions and semi-structured interviews. We took a thematic approach to qualitatively analyze this data.

**Results:**

Our analysis revealed four key challenges: 1) Embracing a holistic, patient centered perspective in a multidisciplinary environment; 2) Decision making under conditions of uncertainty; 3) Balancing the need for patient safety with the need to foster a learning environment for residents; and 4) Taking on a leader’s role and orchestrating an interprofessional team of health care professionals. Newly appointed attendings required extensive practical experience to adapt to their new roles. Most attendings did not receive regular, structured, professional coaching during their transition, but those who did found it very helpful.

**Conclusions:**

Newly appointed attending physician in GIM face a number of critical challenges that are in part specific to the field of GIM. Further studies should investigate whether the availability of a mentor as well as conscious assignment of a series of increasingly complex tasks during residency by clinical supervisors will facilitate the transition from resident to attending.

**Supplementary Information:**

The online version contains supplementary material available at 10.1186/s12909-022-03400-z.

## Background

The transition from resident to attending physician is a major challenge [1]. Attending physicians must master clinical, but also many non-clinical tasks. While postgraduate training focuses on acquiring medical expertise for patient care, many additional non-medical skills needed by an attending, such as leadership, management and teaching are acquired mostly in an informal and unstructured manner during residency [2–4]. The step from supervised clinical care as a resident to independent medical practice as attending physician is correspondingly large. Though several studies of highly specialized medical disciplines have described the transition from resident to attending [5–7], none has elucidated the specific challenges faced by general internal medicine (GIM) attending physicians.

The general internal medicine (GIM) specialty provides comprehensive, high-quality, cost-effective, integrated care for the growing number of mainly elderly patients who have unclear, complex, and/or co-morbid diseases [8–10]. As the number of elderly patients have increased, governments and health care systems have responded to overburdened hospitals by shifting less seriously ill patients from in- to outpatient treatment, shortening hospital stays, and reserving inpatient care for patients who need it most [11, 12]. The needs of these severely ill patients are addressed with the involvement of specialists who have highly specialized knowledge and skills to address specific patient problems. When care from specialists from many disciplines is fragmented and not properly coordinated, this raises costs, extends hospital stays, increases readmission rates, and increases the risk of contradictory treatments [13–16]. Therefore, specialist care for inpatients is often monitored and prioritized by attending physicians in GIM, who bear ultimate responsibility for the optimal care of multimorbid and complex patients. Thus attending physicians in GIM not only must dispose of extensive clinical expertise and knowledge, but also of sound non-clinical skills and experience to manage these patients with a diverse team of health care providers [11].

Newly appointed attendings consider themselves mostly well prepared for clinical tasks, but not for non-clinical tasks [17]. The challenge of mastering these non-clinical skills is regularly described as daunting [17, 18]. Studies that examined transition from resident to attending physician from a non-disciplinary perspective describe it as a longitudinal process which should be embedded in everyday clinical practice [19, 20]. By provision of meaningful learning opportunities physicians are allowed to adapt to the new responsibilities by developing new behaviors [19]. This emphasizes the importance of adapting residency training to the requirements of the practice of attendings. The importance of facilitating the transition from resident to attending is even more important in times of physician shortage [21, 22]. In filling the vacancies, it is not uncommon for advanced residents who have not yet completed their training to be promoted to attending physicians.

Therefore, we designed a qualitative study to identify barriers faced by newly appointed attendings particularly in GIM and facilitators of a successful transition from resident to attending physician.

## Methods

### Study design

Our qualitative exploratory study proceeded from a social-constructivist perspective and explored participants’ views of the challenges and difficulties faced by new attending physicians in GIM and of potential facilitators to meet these challenges. We collected data in focus group (FG) discussions with residents and attending physicians and conducted individual semi-structured interviews with department heads.

Based on our research questions and a preliminary literature search on medical education, learning in the workplace, and transition, we drafted a preliminary question flow and guide for our semi-structured interviews. We adapted the wording of some questions after piloting the guide in a ‘think aloud’ session with residents and attendings at the GIM department of the University of Bern. Participants in the 'think aloud' did not participate in the subsequent course of the study. The questions used in the FG discussions and the interview guide for the semi-structured interviews are reproduced in Appendix A.

### Setting and participants

The study was conducted in Switzerland, where doctors must train for five years to earn a specialist degree in GIM. Training includes three years of basic education in GIM and two years of advanced training in GIM or another subspecialty [23]. Residents are eligible for board certification if they acquire the required competencies, which are based on comprehensive learning objectives created by the Swiss Society of General Internal Medicine (SSGIM).

Physicians from GIM departments of 12 hospitals (two University and ten affiliated teaching hospitals) participated in the study. We purposely selected these departments to reflect the distribution of large and small hospitals across both the German- and French-speaking regions of Switzerland. We intended to account for differences in training and working contexts and to capture cultural and language differences. Participating hospitals gave us leads on potential participants, who we then contacted. After following up on those leads, we enrolled 35 clinicians in the study. Of these, 16 were attendings, 14 were residents, and five were department heads. We deliberately chose the three groups of participants to capture their particular perspective in relation to the research questions and to complement each other. We conducted seven FG discussions (two German and one French resident group; two German, one French and one bilingual attending group) moderated by a member of the research team. Residents and attendings were not mixed in the FG discussions. A second research team member helped moderate the session and took notes. FGs comprised three to six participants and lasted between 60 and 90 min. For logistical and feasibility reasons the five department heads participated in individual semi-structured interviews.

This study was performed in accordance with the ethical standards as stated in the Declaration of Helsinki. All participants provided informed consent. Under institutional regulations, our study was deemed exempted from formal ethical approval.

### Data analysis

FG discussions and interviews were audiotaped and transcribed and we thematically analyzed the transcripts [24]. All six members of the core research team iteratively analyzed data from the FGs and interviews. The data collection was staggered. FGs and interviews were analyzed immediately after their collection. We continued data collection until no further new findings were identified (saturation). After each research team member independently read the transcripts, the group assembled to compare notes and analyze the themes. We took both a deductive and inductive analytical approach. For the deductive approach, we adopted a predetermined set of topics from existing competency frameworks like CanMEDS i.e. *medical expert, teacher and manager* and then applied it to the data [25]. The data clustered around the deductive topics were then analyzed in an inductive approach for emerging themes through a cycle of readings and discussion. For this inductive approach, we used situated learning theory as the sensitizing concept [26]. Situated learning theory proposes that learning takes place through social interaction and connecting prior knowledge with authentic activity. As transition is described as a longitudinal process that is embedded in everyday clinical practice, situated learning theory is particularly well suited for analyzing and describing the process of transition in more detail [27]. We continued the cycle of reading and meeting, discussing the data until we could tell a coherent story about participants’ perceptions of the challenges faced by residents transitioning to attending physicians in GIM.

## Results

Most participants began by mentioning the overriding importance of sound medical knowledge and clinical skills, akin to the description of the *medical expert* role in CanMEDS. This was their decisive criterion for judging the competency of an attending physician in GIM. However, participants differently described the specific knowledge and skills required to best fill this role of attending. Their emphasis and focus varied widely, depending on hospital type and its care mandate. They also described additional non-medical expertise and skills. *teacher* and *manager*, which gave us a more complete picture of necessary competencies. There was substantial overlap between these additional skills and the skills participants thought were most challenging for newly appointed attendings in GIM to acquire. We identified main themes, with themes 1 and 2 relating to the role of *medical expert*, theme 3 relating to *teacher*, and theme 4 relating to *manager*: 1) Embracing a holistic, patient centered perspective in a multidisciplinary environment; 2) Decision making under conditions of uncertainty; 3) Balancing the need for patient safety with the need to foster a learning environment for residents; and 4) Taking on a leader’s role and orchestrating an interprofessional team of health care professionals.

### Embracing a holistic, patient centered perspective by balancing the opinions of different medical specialists and integrating them into a larger whole

The GIM attending is tasked with guiding diagnostic and therapeutic management of often multimorbid patients. To accomplish this task attendings must take the suggestions, opinions, and interests of various experts and specialists into account, set priorities and balance them, while always considering the patient’s concerns and goals.

*“There are recommendations from consultants from other (sub-) specialties… In addition to the patient's request, relatives can also have an opinion or an agenda. And there is an extreme range of examinations, therapies... Who prioritizes that? Or who makes sure that [the patient] gets exactly as much medicine as needed, so that their quality of life improves, but without giving them too much medicine.” (Head of Department 2)*

If attendings are to succeed in GIM, they need broad medical knowledge and should be familiar with current guidelines. Without this knowledge, they cannot rank, prioritize, or argue for or against the suggestions specialists make.

*“And you also need to have broad, sound knowledge to be able to argue [with consultants from other (sub-) specialties].” (Attending 2, focus group 5)*

Key challenges of newly appointed attendings who have to work across disciplines included balancing and integrating the opinions and interests of different stakeholders.

*“Knowing how to integrate all this information […] to be able to balance everyone's opinions […] for the good of the patient, I think that would really be something that I would like to develop [more].” (Attending 4, focus group 2)*

### Decision making and taking ultimate responsibility in conditions of uncertainty

Newly appointed attendings feel that being pressured to make decisions and assuming ultimate responsibility under conditions of uncertainty disrupts their work. There are often no straightforward answers to the complex problems posed by treating typical multimorbid patients hospitalized in GIM. The problem is compounded when guidelines and specialists’ suggestions neglect the needs of individual patients or contradict their wishes.

*“The difference between... [the GIM attending] and specialists is that the [GIM attending] has to endure uncertainty. The specialists cannot stand uncertainty. They continue looking to no end.” (Head of Department 1)*

During residency, trainees follow their supervising (attending) physician’s instructions. Although they learn to discuss a patient's problems with specialists, their role in decision making is more passive.

*“And, as a resident [when you didn't know what to do], you simply asked [your attending] and waited for the answer.” (Attending 6, focus group 6)*

Newly appointed attendings must make decisions and assume responsibility, which helps them learn and embrace their new role. They benefit from discussing difficult cases and situations with experienced peers. These conversations foster their ability to make decisions under uncertain conditions and to trust their own judgment.

*“But that certainly made me grow, that the responsibility was also transferred. Of course, you always get help from your colleagues and ask if you made the right decision or not.” (Attending 7, focus group 6)*

A small minority of attending physicians struggle with making decisions and assuming responsibility. Even though their colleagues usually think them being knowledgeable, they labor under the burden of their own high expectations. The need for self-efficacy is most obvious when it is absent.

*“These [attendings] were from a medical expert perspective... absolutely up to date. But somehow …they simply did not have enough confidence themselves [in their own competence]. » (Head of department 2)*

Residents are put in a difficult position by attendings who are troubled by uncertainty and the burden of responsibility for their decisions, especially when attendings change their minds repeatedly or postpone decisions.

*“What I hated were attendings who did not have a clear vision [about patient care], but who changed their mind each time.” (Resident 2, focus group 3)*

### Finding a balance between guaranteeing patient safety and creating a learning environment for residents

Responsibility for patient safety is closely associated with supervising residents. The attending must have an overview of patient care to guarantee safe and good practice, but must balance this carefully with their residents’ need for enough autonomy to encourage professional development.

*“… supervision is…, on the one hand, allowing the resident to develop, but on the other hand, having sufficient control over patient care.” (Attending 4, focus group 7)*

Newly appointed attendings often tilt the balance toward patient safety and are reluctant to delegate to and trust in the residents’ abilities.

*“This [trusting in residents] is also extremely hard for me. Because you still have the feeling that, when I do it myself, then I know that it’s done.” (Attending 1, focusgroup 5)*

As they gain experience, attendings can increasingly grant their residents autonomy by letting them learn by experience and helping them thrive by creating a learning environment.

*“It was to find the right balance between the right degree of supervision, to be close enough to the resident and direct patient care,… but at the same time let them [the residents] a little autonomy, a little air to breathe... “ (Attending 3, focus group 2)*

When an attending offers more autonomy to residents who are ready for it, it helps the residents to become more competent.

*“The attending who was able to step back… I appreciate extremely…. for example, that they could say, ‘I would probably have done it differently now, but it still works.’” (Resident 5, focus group 4)*

Attendings may not feel adequately prepared to assume the demanding role of a teacher who takes responsibility for their residents’ development.

*“… you are a teacher. You should teach, you should be strong in communication. You should be able to give constructive feedback. And you just ask yourself, where should I have learned this? It was never taught in medical school.” (Attending 6, focus group 6)*

### Taking on a leader’s role and orchestrating an interprofessional team of health care professionals

To manage hospitalized multimorbid patients, an attending must solicit the input of many different health care professionals. The attending must lead and manage an interprofessional team of medical specialists, residents, students, nurses, physical therapists, psychotherapists, nutritionists, and social service workers. The attending directly supervises residents, but other team members may operate in adjacent hierarchies. To orchestrate the interests and needs of the various experts involved in heterogeneous teams, the attending must develop skills in negotiation and diplomacy.

*“The position of the attending is a classic sandwich, where you are, so to speak, sandwiched between the head of department, the patient, the resident, the nursing staff and the consultants from other specialties.” (Head of Department 2)*

The attending who leads the team also becomes its main troubleshooter. It is the attending’s job to solve a broad spectrum of problems, including resolving disagreements between the ward’s staff and the patients or organizational tasks.

*“… suddenly you are responsible for everything. So the nursing staff has a dispute with the patient or the resident and then the attending has to sort it out. Simply, all things that don't work, including organizational matters, ultimately come to the attending.” (Attending 3, focus group 6)*

## Discussion

Our results highlight four key themes that newly appointed attending physicians in GIM most often struggle with: 1) Embracing a holistic perspective in a multidisciplinary environment; 2) Decision making under conditions of uncertainty; 3) Balancing the need for patient safety with the need to foster a learning environment for residents; and 4) Taking on a leader’s role and orchestrating an interprofessional team of health care professionals.

The challenges we identified in the transition from resident to newly appointed attending suggest that achieving competence is best understood as a process [28] that ideally begins in residency. In line with findings of other transition studies, we found that the attendings in GIM are challenged by their new responsibilities as a team leader and teacher [17, 18]. The issue in relation to the role of teacher and leader are specific to GIM and are closely intertwined with the multidisciplinary environment (theme 1). Attendings in GIM must do more than master clearly defined tasks. While many medical disciplines focus on diagnosing the cause of a clearly defined problem and providing appropriate treatment, GIM poses an additional challenge, best shown by a typical case: *an older, multimorbid patient with asthenia is moderately enthusiastic about the therapy for his chronic diseases, but the conduct of his relatives imply that the patient has not been receiving the best medical care lately. This patient was just diagnosed with a new malignant tumor and the treatment prospects are uncertain. The consulting oncologist has told the patient that he might be eligible for inclusion in a clinical trial.* The attending physician in GIM is embedded in a complex system [29–31] of interacting elements. The *patient’s preferences* is only one element, the *relatives* another, the multiple *diagnoses* yet another, and so on. To make a full inquiry within such a system and to coordinate the team in the treatment of the patients, the GIM attending must be able to recognize all the individual elements and also take a systemic view [32]. The particular challenge is that the newly appointed attending has to learn how to lead a large number of different teams which are characterized by their diverse and constantly changing compositions (theme 4). Even apparently identical situations with comparable elements (patient, new malignant tumor, consulting oncologist, relatives, etc.) will differ because relationships between the elements differ. It is in the nature of such complex systems that decisions must often be taken in the context of uncertainty (theme 2). Taking decisions and bearing the ultimate responsibility in such a situation is already a big challenge for the newly appointed attending. Attendings though not only have the responsibility to make the right medical decisions but also share the responsibility for the learning progress of their residents. Newly appointed attendings are both stressed and distressed when faced with the need to integrate residents in patient care in a purposeful way, so that they are optimally supported in their competence development, while at the same time not threatening patient safety (theme 3). The teacher role in GIM is thus quite different from the teacher role in other medical specialties. It is one thing to supervise a resident in clearly defined tasks such as technical procedures where the conditions can be defined and controlled. It is quite another to provide the right amount of supervision in a dynamically interacting multidimensional environment such as the care of multimorbid patients. Attending physicians in GIM must learn how to navigate in this complex, multidimensional environment. In order to better understand how to support the transition from resident to attending, we further evaluated our findings by examining them through the lens of situated learning theory.

In situated learning theory, the resident is framed as a member of the physician team that ‘co-participates’ in and learns from daily clinical activities [22]. Residents and attendings function within social structures (communities of practice) embedded in power relations. When leading a team and taking decisions about patient care, attendings determine whether a resident’s peripheral participation is an ‘empowering’ or ‘disempowering’ experience [27]. The residents may or may not be given more responsibility and higher stakes tasks that teach them to make decisions in uncertain conditions. Encouragement to perform actively and constructive feedback given by attendings could support the residents in their transition to a more central role, taking comprehensive clinical responsibility. Competence and self-confidence can be safely gained if the resident’s clinical supervisor closely monitors the resident’s development and purposefully delegates tailored assigned tasks appropriate to the resident’s level of training. Such an approach to training would imply that learning is not merely a by-product of work (in the context of legitimate peripheral participation), but also includes learning opportunities that are deliberately directed. This process would be akin to the notion of ‘guided participation’ where tasks or activities are intentionally selected by supervisors to facilitate learning [33].

Our study results indicate that the learning environment is key for residents to develop decision-making skills and to learn navigate in a multidimensional, complex environment. Provision of such a learn-stimulating environment is—at the same time—also a major challenge for newly appointed attendings as they have to balance it with patient safety issues. New attendings would benefit from a designated mentor who provides advice and support at regular intervals, helping them mature in their new role [34]. Such measures to facilitate transition are all the more important in times of physician shortage when advanced residents who have not yet completed their training are promoted to attending physicians.

Our study has certain limitations. Postgraduate training shapes the attendings’ perceptions of their transition from resident [35], so our results may not be generalizable beyond Switzerland because specialist training in GIM and the exact duties of an attending physician differ across countries and different health care systems. Our study, however, is embedded within a clearly defined conceptual framework and thus our findings have sound theoretical explanation and seem to be fundamental, rather than representing a specific local phenomenon.

## Conclusions

Attending physicians in GIM face a number of challenges when newly appointed to this position. Major challenges include decision making under uncertainty in a multidisciplinary setting, the task of balancing the need for patient safety with the need to foster a learning environment for residents, and orchestrating an interprofessional team. Transition from resident to attending is best seen as a longitudinal process. If residents are to successfully transition to attending physicians, they should not simply be expected to learn by experience. Instead, their clinical supervisor should consciously assign them a series of increasingly complex tasks to develop their decision making proficiency and self-confidence, and each resident should be assigned a mentor who provides regular advice and support.

## Supplementary Information

**Additional file 1.**

## Data Availability

Datasets analysed during this study are available from the corresponding author on reasonable request.

## Abbreviations

FG: Focus group
GIM: General Internal Medicine
SSGIM: Swiss Society of General Internal Medicine
