# Supplementary material for: Challenges in the transition from resident to attending physician in general internal medicine: a multicenter qualitative study
Source: BMC Med Educ. 2022 May 2;22:336. doi: 10.1186/s12909-022-03400-z (PMC9063076; doi:10.1186/s12909-022-03400-z)
Supplement: Supplementary file 1 — Additional file 1. [file 12909_2022_3400_MOESM1_ESM.docx]

**Appendix A**

Question Routes and interview guides

Residents

1) What makes / would make you most anxious if you (would) take on the position of an attending physician?

2) Can you describe attending physicians who do their job particularly well?

a. In what specific aspects of their work do they perform particularly well?

3) Can you describe attending physicians who are more likely to have difficulties with their work?

a. Which specific aspects of their work make them struggle the most?

4) Which competencies seem to provide the most difficulties to attending physicians?

a. Are there differences between younger / more experienced attending physicians?

5) What should be done specifically to prepare (future) attending physicians as well as possible for their task?

6) To what extent have you been supported in your career planning / did you have any individual mentoring?

7) What are the most important competencies for an ideal attending physician in General internal medicine (GIM)?

If no answer:

a. Hospital-relevant professional and content-related skills (knowledge, technical skills)

b. see Core Competencies in Hospital Medicine

Attending Physicians

1) In which specific aspects of your work as an attending physician did you feel confident / competent from the start?

a. What was this security / competence based on?

2) With which aspects of the work did you struggle the most as a newly appointed attending physician?

a. Who and what supported you in this situation?

b. How did you acquire the necessary skills?

c. Has anyone had any experience with mentoring?

3) In which aspects of your work as an attending physician have you learned the most since you started working as an attending physician?

In which fields do you feel that you should acquire additional skills in order to meet your ideal of an attending physician?

4) What is this situation like for your colleagues?

With Which aspects of their work do your colleagues struggle the most?

Do you have specific examples?

5) For those of you who have already worked as an attending physician at another hospital: To what extent do the competencies required for an attending physician in GIM differ across hospitals?

6) If additional training / courses (which?) have been completed; to what extent were these helpful for your work?

7) What specifically should be done to ensure that future attending physicians are prepared as well as possible for their task?

8) What are the most important skills for an ideal attending physician in GIM?

If no answer:

a. Hospital-relevant professional and content-related skills (knowledge, technical skills)

b. see Core Competencies in Hospital Medicine

Head of departments

1) How do you select residents who you later want to hire / promote as attending physicians?

a. What criteria is this selection based on?

b. Have you already experienced (pos / neg) surprises here? (ask for a specific example)

2) Can you describe attending physicians who do their job particularly well?

a. Which specific aspects of the work does the person concerned do particularly well?

3) Can you describe attending physicians who are more likely to have difficulties with their work?

a. Which specific aspects of their work make them struggle the most?

b. How did you deal with this situation?

c. What exactly have you done to support the person concerned?

4) What was your experience of hiring an attending physician who had already worked in a same position at another hospital

a. To what extent do the competence requirements for an attending physician GIM differ from hospital to hospital?

5) In which field of competence do attending physicians currently have the greatest deficits

a. Are there differences between younger / more experienced attending physicians?

6) What should be done to prepare future attending physicians as well as possible for their task?

7) What are the most important competencies for an ideal hospital attending physician at GIM ?

If no answer:

a. Hospital-relevant professional and content-related skills (knowledge, technical skills)

b. see Core Competencies in Hospital Medicine
